# Thoracic spondylotic myelopathy in diffuse idiopathic skeletal hyperostosis: a comparative study

**DOI:** 10.1186/s13018-023-03723-7

**Published:** 2023-03-25

**Authors:** YuLei Dong, Jiahao Li, Kaili Yang, Shigong Guo, Jiliang Zhai, Yu Zhao

**Affiliations:** 1 Department of Orthopaedic Surgery, Peking Union Medical College Hospital, Peking Union Medical College and Chinese Academy of Medical Science, No. 1 Shuaifuyuan Dongdan, Dongcheng District, Beijing, 100730 People’s Republic of China; 2 Eight-Year Medical Doctor Program, Chinese Academy of Medical Sciences & Peking Union Medical College, Beijing, People’s Republic of China; 3 Department of Rehabilitation Medicine, Southmead Hospital, Bristol, UK

**Keywords:** Thoracic spondylotic myelopathy, Diffuse idiopathic skeletal hyperostosis (DISH), Complications, Surgery, Thoracic spinal stenosis

## Abstract

**Background:**

To explore the characteristics and clinical management of thoracic spinal stenosis with diffuse idiopathic skeletal hyperostosis (DISH).

**Methods:**

The patients diagnosed with thoracic spondylotic myelopathy who underwent spinal decompression and fusion surgery in a single center between 2012 and 2020 were retrospectively analyzed. All the patients were followed up for at least 2 years. Patients were classified into DISH and non-DISH groups. Demographic, radiographic and clinical parameters were compared between the two groups.

**Results:**

A total of 100 thoracic spondylotic myelopathy patients were included in the study. 22 patients were diagnosed with DISH. The proportion of male patients in the DISH group was higher, and the average BMI was larger. The incidence of upper thoracic vertebrae with ossification of posterior longitudinal ligament (OPLL) (*P* < 0.05) and lumbar spine with ossification of ligamentum flavum (OLF) was higher (*P* < 0.05) in DISH the group. The proportion of patients received staged surgery is higher in the DISH group (*P* < 0.1). There were no significant differences between the two groups in the amount of surgical bleeding, the ratio of cerebrospinal fluid leakage, the time duration of drainage tube placement and the JOA scores.

**Conclusion:**

Thoracic spinal stenosis with DISH occurred more in male patients with larger BMI. The posterior decompression and fusion surgery could achieve comparable satisfying clinical outcomes between DISH and non-DISH patients. More proportion of patients received staged surgery in the DISH group; the underline mechanism may be DISH caused more OPLL in the upper thoracic spine and more OLF in the lumbar spine because of mechanical stress.

## Introduction

Thoracic spinal stenosis (TSS) is a disease in which compression of the thoracic spinal cord and nerve roots leads to the corresponding clinical signs and symptoms. TSS could lead to numbness and even paralysis in the lower extremities, disturbance of bladder and bowel, and others. TSS is mainly caused by ossification of ligamentum flavum (OLF), ossification of posterior longitudinal ligament (OPLL), and disc herniation (DH) [1]. While diffuse idiopathic skeletal hyperostosis (DISH) is a disease characterized by the presence of more than three bony bridges resulted from ossification of the anterior longitudinal ligament. Spinal flexibility is reduced by continuous bony bridges and concentration of mechanical stress occurs on the boundary between ossified and unfused vertebrae body. This can lead to OLF, OPLL, and DH [2], causing various thoracic stenosis and spinal cord compression. However, the clinical characteristics of thoracic spinal stenosis in DISH are unclear.

In vertebral fractures with DISH, the fused vertebra has been found to act as a long lever arm, giving rise to stress concentration at the fracture site [3]. Therefore, we speculate that the stress between fusion and adjacent segments in DISH group is greater than that in non-DISH group, and similar manifestations of adjacent segment degeneration will occur. TSS patients with and without DISH may have different clinical characteristics.

This study retrospectively analyzed thoracic stenosis patients who underwent spinal decompression fusion at a single center from 2012 to 2020. All the patients underwent at least 2 years of follow-up. The hypothesis was DISH could cause thoracic spinal stenosis with specific characteristics compared with non-DISH patients.

## Materials and methods

### Inclusion and exclusion criterion

All the patients with the diagnosis of thoracic spinal stenosis who underwent thoracic spinal decompression and fusion surgery in Peking union medical hospital (2012-2020) were retrospectively analyzed. They were divided into DISH group and non-DISH group based on whether they were diagnosed as DISH or not. DISH was diagnosed by Resnick’s [4] diagnostic criteria. (i) Bony bridges were found on the anterior or lateral of more than three consecutive vertebral bodies. (ii) There is a relatively preserved intervertebral disc space; (iii) No sacroiliac joint ankylosis. All the above observations are based on sagittal images of computed tomography (CT).

Patients who had been followed up for less than 2 years were excluded. Patients with incomplete recorded of demographic information, diagnosis, surgical notes and charts, and complications were also excluded.

### Surgical process

All operations were performed by the same senior spinal surgeon. After general anesthesia, the patient was placed in the prone position, paravertebral muscles were stripped along the posterior median of the spine, and pedicle screws were placed. A high-speed grinding drill was used to notch along both sides of the vertebral lamina and grind the vertebral lamina to thinness. A Kerrison lamina rongeur was then used to remove the lamina in one piece or several pieces. Any ossified dura was also removed. The deep fascia and skin were closed and sutured after drainage. The drainage tube was removed 72 hours post-surgery. If there was cerebrospinal fluid leakage, the drainage tube should be removed within 1 week.

### Parameters analyzed

All patients underwent the whole spine X-rays before the operation, immediately after surgery, and at 3 months, 6 months, 1 year, 3 years, and 5 years after the surgery. The demographic parameters, radiographic parameters, surgical parameters, and preoperative, postoperative and follow-up Japanese Orthopedic Association (JOA) scores were recorded.

### Statistics

For continuous variables, independent sample T test was used. For categorical variables, Chi-square tests or Fisher exact tests are used. *P* < 0.05 was considered a statistically significant difference in this study.

## Results

**Fig. 1:**
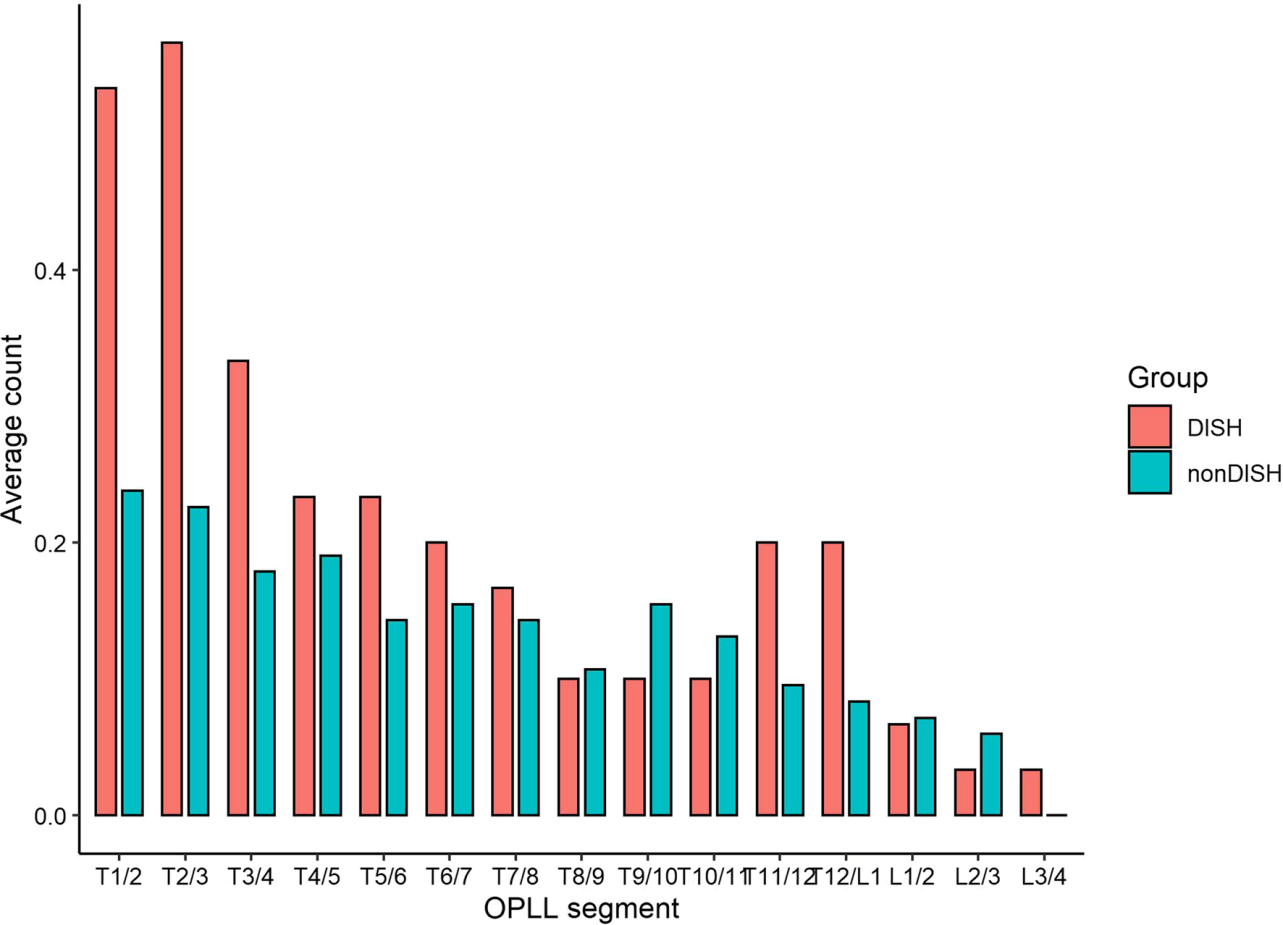
Proportion of OPLL in each segment of DISH group and non-DISH group

**Fig. 2:**
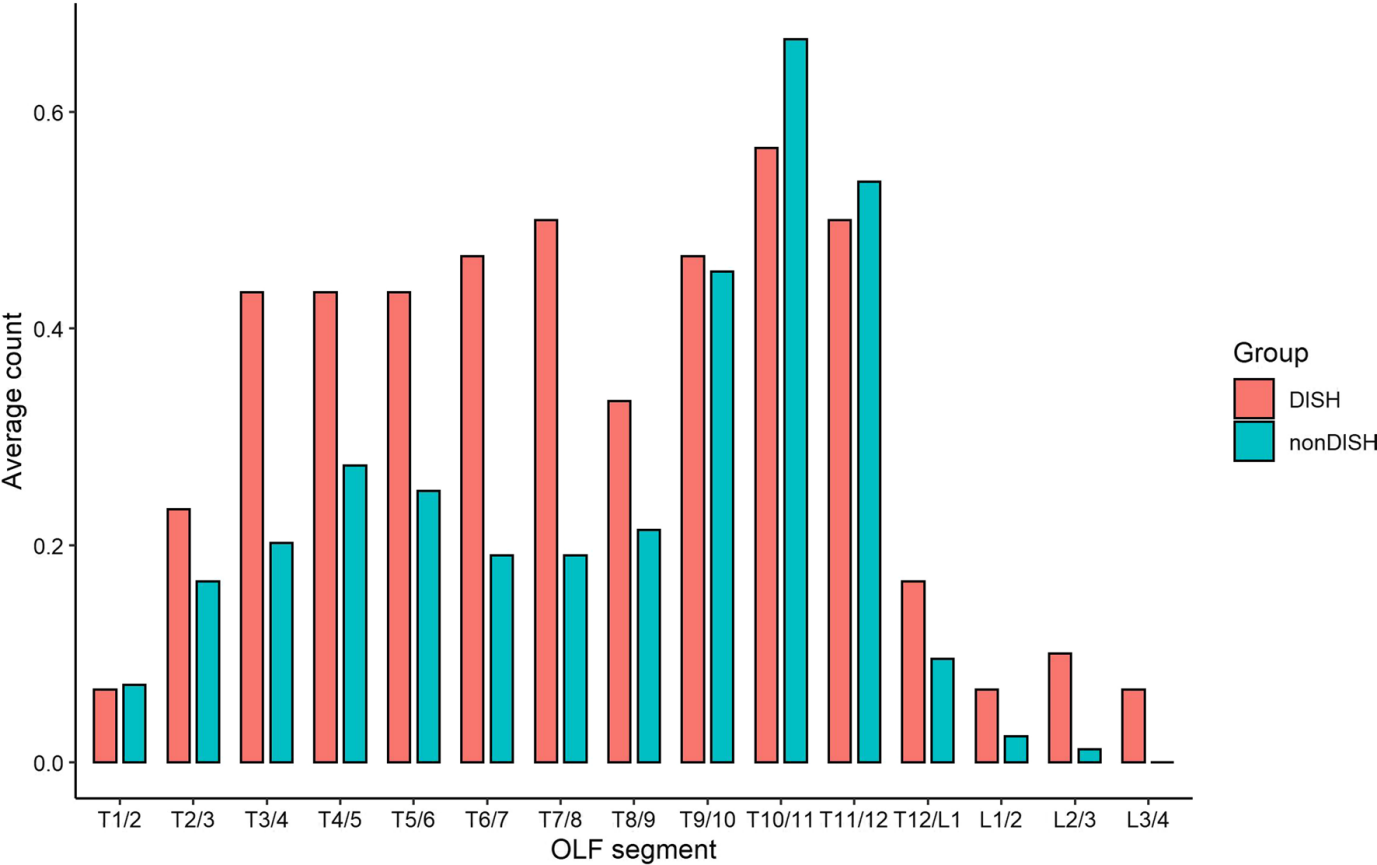
Proportion of OLF in each segment of DISH group and non-DISH group

**Fig. 3:**
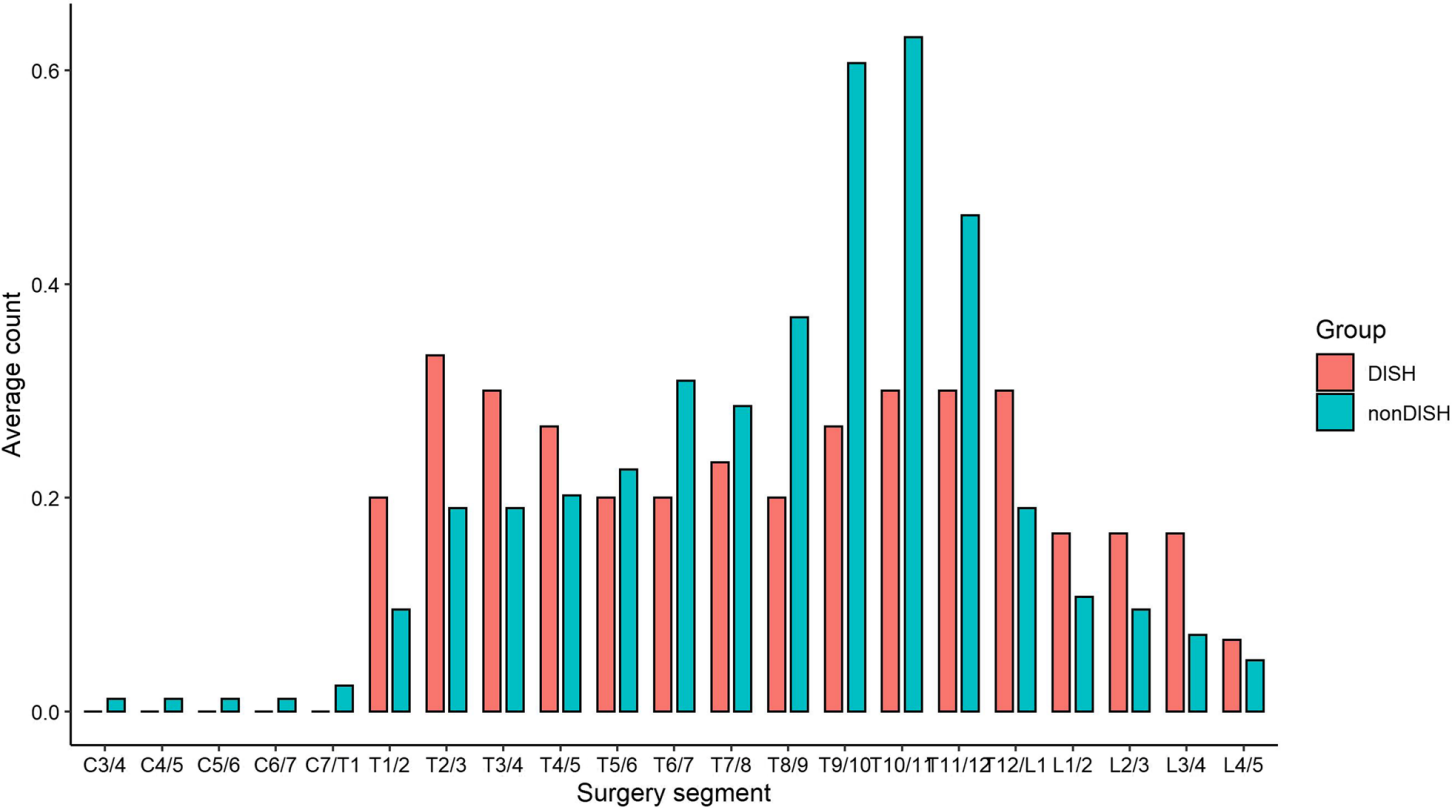
Segment distribution of surgical site between DISH and non-DISH group

**Fig. 4:**
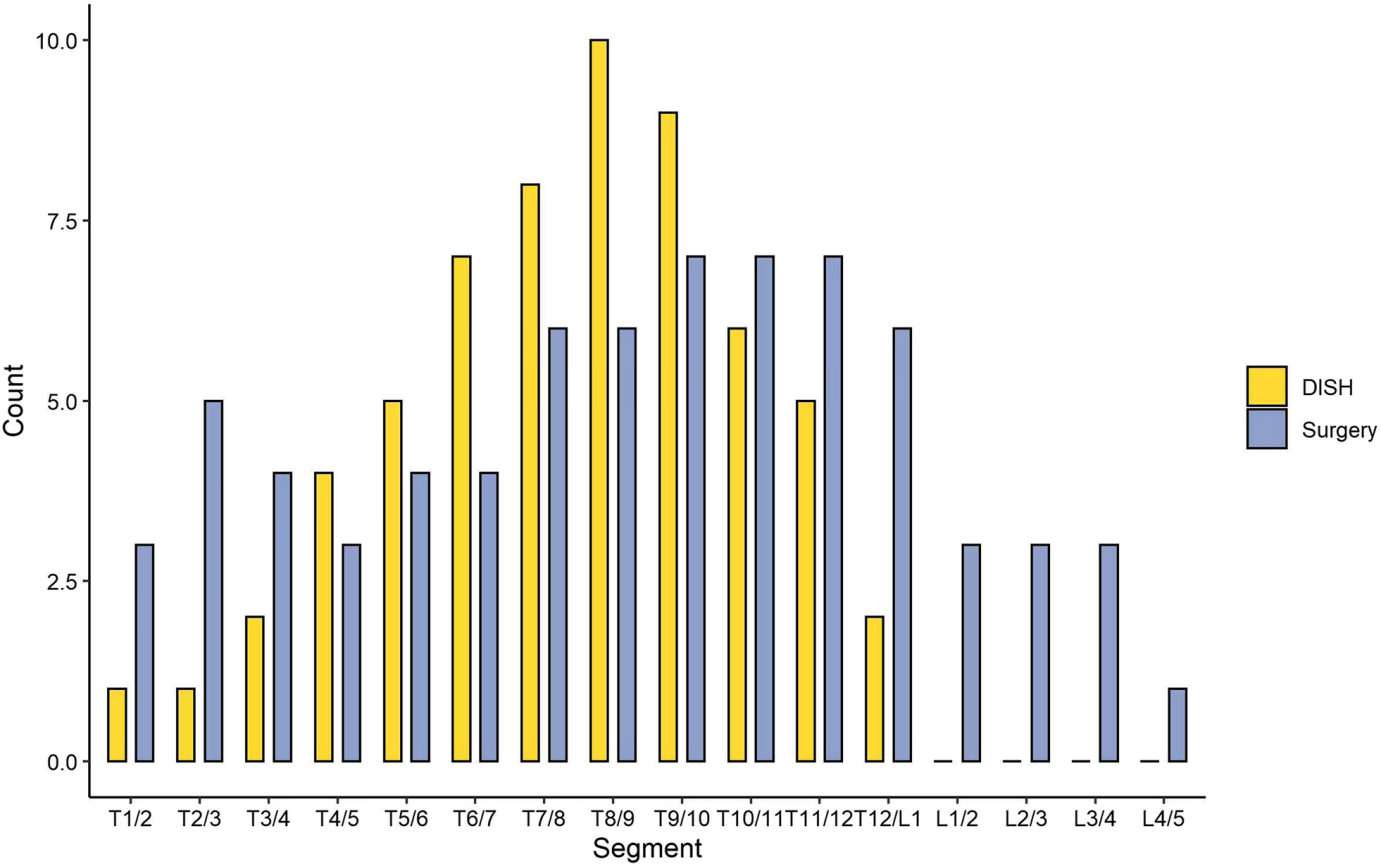
Anterior longitudinal ligament ossification segment and surgical decompression segment in DISH group

Among 140 consecutive cases of thoracic spinal stenosis who underwent spinal decompression surgery, 100 patients were enrolled. 22 cases were combined with DISH, and 78 cases were non-DISH. There was no significant difference in age and comorbidity between the DISH and non-DISH groups. There was a trend, although not statistically significant, the proportion of male patients in the DISH group was higher, and the average BMI was greater (see Table 1). The incidence of thoracic vertebrae with OPLL in the DISH group was higher (63.6% vs 38.5%, *P* < 0.05), and more OPLL was distributed in the upper thoracic segment (Fig. 1). The incidence of lumbar ligamentum flavum ossification was higher in the DISH group (31.8% vs 12.8%, *P* < 0.05) (see Table 2). The lesions at the responsible segment leading to compression were mostly caused by the OLF in both groups. The stenotic segments in the DISH group were more evenly distributed, so a higher proportion of patients received staged surgery (Figs. 2, 3, and Table 3). However, in the non-DISH group, the stenosis segment was more concentrated in the thoracolumbar segment, and the surgical segment was also concentrated here. In DISH patients, there was a correlation between the segment site of surgical decompression and the ossification segment of the anterior longitudinal ligament (Fig. 4). No statistical difference was found between the two groups in the amount of surgical bleeding, the rate of cerebrospinal fluid leakage, and the duration of drainage tube placement. There was no statistical difference in JOA scores as well.

**Table 1:** Demographic characteristic and comorbidity between DISH and Non-DISH groups

Characteristics	DISH (*n* = 22)	Non-DISH (*n* = 78)	*P* value
Age (mean, SD)	53.2 ± 11.6	52.9 ± 10.8	0.91
Gender			0.06
Male (percent)	14 (63.6)	30 (38.5)	
Female (percent)	8 (36.4)	48 (61.5)	
BMI (mean, SD)	29.8 ± 5.7	27.2 ± 4.6	0.015
Comorbidity			
HTN (percent)	7 (31.8)	21 (26.9)	0.94
DM (percent)	4 (18.2)	7 (9.0)	0.28
CSM (percent)	8 (36.4)	25 (32.1)	0.98
LSS (percent)	11 (50.0)	38 (48.7)	1

HTN, hypertension; DM, diabetes mellitus; CSM, cervical spondylotic myelopathy; LSS, lumbar spinal stenosis

**Table 2:** Radiographic characteristics comparison between the two groups

Characteristics	DISH (*n* = 22)	Non-DISH (*n* = 78)	*P* value
OPLL (percent)	14 (63.6)	35 (44.9)	0.12
C-OPLL (percent)	14 (63.6)	35 (44.9)	0.12
T-OPLL (percent)	14 (63.6)	30 (38.5)	0.04
L-OPLL (percent)	6 (27.3)	10 (12.8)	0.10
OLF (percent)	21 (95.5)	75 (96.2)	1
C-OLF (percent)	1 (4.5)	2 (2.6)	0.53
T-OLF (percent)	21 (95.5)	74 (94.9)	1
L-OLF (percent)	7 (31.8)	10 (12.8)	0.04
PID (percent)	8 (36.4)	20 (25.6)	0.32

OPLL, ossification of posterior longitudinal ligament; C, cervical; T, thoracic; L, lumbar; OLF, ossification of ligamentum flavum; PID, prolapsed intervertebral disc

**Table 3:** Surgical parameters comparison between the two groups

Characteristics	DISH (*n* = 22)	Non-DISH (*n* = 78)	*P* value
Responsible lesion			0.33
OPLL (percent)	7 (31.8)	17 (21.8)	
OLF (percent)	15 (68.2)	61 (78.2)	
Staged-surgery (percent)	8 (36.4)	14 (17.9)	0.07
Post-operational manifestation			
Blood loss (mean, SD)	1166 ± 871	912 ± 681	0.11
CSF leakage (percent)	11 (50.0)	35 (44.9)	0.97
Drainage time (mean, SD)	5.4 ± 2.6	5.3 ± 2.7	0.94
JOA score-preop (mean, SD)	10.0 ± 3.2	10.9 ± 2.6	0.13
JOA score-postop (mean, SD)	11.7 ± 3.2	12.5 ± 2.8	0.22
JOA score-follow up (mean, SD)	13.7 ± 3.1	14.4 ± 3.1	0.28

OPLL, ossification of posterior longitudinal ligament; OLF, ossification of ligamentum flavum; CSF, cerebrospinal fluid

## Discussion

TSS is a relatively rare disease, its etiology and mechanism are complex, and its early diagnosis and treatment are very difficult. These challenges result in the worsening of neurological symptoms and poorer prognosis that are closely associated with disease progression [5]. However, recent research on the etiology, early screening and diagnosis of thoracic stenosis is gradually revealing the progression of TSS. Stimulation of mechanical stress may be one of the driving factors [6, 7]. Quantitative measurement by CT or Magnetic Resonance Imaging (MRI) can help us to predict the operative risk and postoperative recovery of TSS to a certain extent [8]. In clinical work, some TSS patients are diagnosed with combined DISH. DISH is a systemic disease and its characteristics can be summarized as continuous ossification of ligaments and entheses, especially in the axial skeleton. A Japanese study showed that the prevalence of DISH was 10.8%(22.0% in male and 4.8% in female), which was higher in the elderly, based on a cohort study of the population [9]. However, no age difference was found between patients with and without DISH in TSS in this study. This may be explained by the fact that the onset age of TSS is close to that of DISH. Most DISH patients are considered asymptomatic except back pain and stiffness [10], resulting in the high difficult to study the natural history and characteristics of DISH. Nevertheless, DISH can present with significant clinical consequences, containing spinal fractures due to low-energy trauma and dysphagia or airway obstruction due to anterior cervical pontine osteophytes [11, 12]. Pathological changes in DISH patients result in the loss of the effect of tissues that buffer stress, such as the intervertebral disc, and stress concentration in the patient’s spine. Patients with DISH also have an increased incidence of OPLL and OLF, both of which can cause spinal stenosis [13]. However, as a possible cause of TSS, the disease characteristics of TSS in patients with DISH are not yet clear.

In this study, among 100 cases of thoracic spinal stenosis, 22 cases were combined with DISH. Some reports indicated that DISH is associated with thoracic spondylotic myelopathy, suggesting that DISH is a risk factor for the development of thoracic spondylotic myelopathy. Takagi et al. reported a case of thoracic spondylolisthesis in which the lesion appeared at the upper and lower boundaries of the bony bridge without ossification in the spinal canal [14]. Yamada et al. indicated that chronic pressure in the intervertebral space at the unfused segments on the both ends of DISH lesions was one of the risk factors for the development and exacerbation of lumbar stenosis, and that a greater proportion of DISH patients required surgery [15]. The thoracic vertebrae are not as mobile as the lumbar vertebrae, but the increased mechanical stress caused by DISH may lead to disc degeneration and hypertrophy or ligamentum flavum hypertrophy, and further progression to TSS.

In this study, the proportion of male in DISH group (63.6%) is also higher than that in non-DISH group (38.5%), and the BMI was greater, which correlated with the current literature [16, 17]. This suggests that both male and obesity may be risk factors for TSS combined with DISH. It is reported that DISH patients has a higher probability of developing into thoracic spondylotic myelopathy due to degeneration caused by increasing mechanical stress between the bony bridges and the adjacent healthy vertebral [18]. Hou et al. reported that the lower thoracic stenosis was caused by OLF in 56% of patients, while the upper thoracic stenosis was caused by OPLL in 90% of patients [19]. In this study, we found a higher incidence of concomitant thoracic OPLL and lumbar OLF in the DISH group. This study found that OPLL occurred more frequently in the upper thoracic segments in the DISH group. This is consistent with the main etiology of upper thoracic stenosis reported. OLF mainly occurred in the thoracolumbar region in the non-DISH group. Although the mechanism that causes segmental specificity in TSS patients with DISH is not fully understood, mechanical stress is thought to be a key factor. As the transition regions of spinal curvature, the upper thoracic and lower thoracic regions are more likely to degeneration under constant and strong mechanical pressure stimulation [20].

The bony bridges in DISH patients are diffuse, resulting in the characteristics of the compressed segment of the spinal cord that are different from the local compression in non-DISH patients. Therefore, the selection of surgical sites is evenly distributed, rather than showing a tendency to concentrate in the thoracolumbar segment. Although patients diagnosed with DISH had some distinct characteristics, the surgical outcomes were similar compared with non-DISH patients. After the decompression and fusion surgery, the JOA scores at follow-up improved compared with the preoperative condition. However, as the DISH often involved the middle and lower thoracic spine, the responsible lesions were often septal, and thus staged surgery should be performed [21]. However, DISH has been identified as a risk factor for unanticipated second surgery in patients with lumbar degenerative disease, with a revision rate of up to 19% in DISH patients versus 6.9% in non-DISH patients [22]. The sample size in this study was limited and no revision data for TSS were observed. The incidence of postoperative long-term complications in TSS patients with DISH deserves continuous attention. Although no difference in postoperative outcomes was found between DISH and non-DISH TSS patients in this study. We should consider TSS with DISH as a disease with a higher risk, because of the focal segmentary characteristics of TSS patients in the DISH group and the fact that multi-segmentary stenosis is common in DISH patients and may require staging surgery.

This study has the following shortcomings. First, as thoracic spinal stenosis combined with DISH was a relatively rare disease, so the number of cases was limited. Second, this was a retrospective study. However, all the surgical data and JOA scores were collected prospectively and all the surgeries were performed by the same spine surgeon group which ensured consistency of treatment. In addition, the ossification break in DISH is also a concern. The resulting appearance of OPLL/OLF and surgical site may also be different from that of common TSS patients. However, the cases of DISH included in this study were most isolated bridging of anterior ligament. So ossification breaks could not be found in these cases. Further studies are needed about this question.

## Conclusions

Thoracic spinal stenosis with DISH occurred more in male patients with larger BMI. DISH caused more OPLL in the upper thoracic spine and more OLF in the lumbar spine. The posterior decompression and fusion surgery could achieve satisfactory outcomes and the clinical outcome and surgical complications were comparable between DISH and non-DISH patients. A greater proportion of patients received staged surgery in the DISH group.

## Data Availability

The datasets used and/or analysed during the current study are available from the corresponding author on reasonable request.

## Abbreviations

DISH: Diffuse idiopathic skeletal hyperostosis
BMI: Body mass index
OPLL: Ossification of posterior longitudinal ligament
OLF: Ossification of ligamentum flavum
TSS: Thoracic spinal stenosis
DH: Disc herniation
